# A new *Gephyromantis* (*Phylacomantis*) frog species from the pinnacle karst of Bemaraha, western Madagascar

**DOI:** 10.3897/zookeys.81.1111

**Published:** 2011-02-18

**Authors:** Angelica Crottini, Frank Glaw, Maurizio Casiraghi, Richard K.B. Jenkins, Vincenzo Mercurio, Christian Randrianantoandro, Jasmin E. Randrianirina, Franco Andreone

**Affiliations:** 1 Division of Evolutionary Biology, Zoological Institute, Technical University of Braunschweig, Spielmannstr. 8, 38106, Braunschweig, Germany; 2 Sezione di Zoologia e Citologia, Dipartimento di Biologia, Universita´ degli Studi di Milano, Via Celoria 26, 20133 Milano, Italy; 3 Zoologische Staatssammlung München, Münchhausenstraße 21, 81247 München, Germany; 4 ZooPlantLab, Universitá degli Studi di Milano-Bicocca, Dipartimento di Biotecnologie e Bioscienze, Piazza della Scienza 2, 20126 Milano, Italy; 5 Madagasikara Voakajy, BP 5181, Antananarivo 101, Madagascar; 6 Durrell Institute of Conservation and Ecology, School of Anthropology and Conservation, University of Kent, Canterbury, CT2 7NR, UK; 7 Museum für Naturkunde, Leibniz-Institut für Evolutions- und Biodiversitätsforschung an der Humboldt-Universität zu Berlin, Invalidenstraße 43, D-10115 Berlin, Germany; 8 Parc Botanique et Zoologique de Tsimbazaza, BP 4096, Antananarivo 101, Madagascar; 9 Museo Regionale di Scienze Naturali, Via G. Giolitti 36, 10123 Torino, Italy

**Keywords:** Amphibia, *Gephyromantis atsingy* sp. n., Madagascar, Tsingy de Bemaraha

## Abstract

We describe a new mantellid frog of the subfamily Mantellinae from the karstic Bemaraha Plateau, western Madagascar. The new species belongs to the genus Gephyromantis, subgenus Phylacomantis, which previously included Gephyromantis azzurrae, Gephyromantis corvus and Gephyromantis pseudoasper. Gephyromantis atsingy **sp. n.** has a snout-vent length of 35–43 mm and is a scansorial frog living among the Tsingy de Bemaraha pinnacles and inside the caves present in the area. A morphological analysis and biomolecular comparison revealed the degree of differentiation between these four species of the Phylacomantis subgenus.The new species seems to be endemic to Tsingy de Bemaraha.

## Introduction

The intense herpetological activity carried out in Madagascar during the last decades, together with the wider use of integrative taxonomic tools has led to the description of an astonishingly high number of new amphibians species (Köhler et al. 2005, Vences et al. 2008, Glaw et al. 2010) and to the identification of numerous still undescribed candidate species (Vieites et al. 2009).

Although, the highest species richness of amphibians is typically found along the eastern rainforest belt (Andreone et al. 2005, Glaw and Vences 2007), an increasing number of peculiar species are known from the arid western part of Madagascar (Glaw et al. 1998, 2006, Glos et al. 2005, Mercurio and Andreone 2007, Bora et al. 2010). At these sites the research effort has been gradually increased in recent years, and systematic surveys have recently taken place (e.g. Mercurio et al. 2008, Raselimanana 2008, Bora et al. 2010).

While the species already described from the arid West mostly belong to radiations of explosive breeders reproducing in ephemeral ponds, a special attention has been given to species ascribed to clades that are more typical of humid habitats and rainforest biomes. This was the case, for example, with the recent discovery of two new mantellines at the Isalo Massif (Mercurio and Andreone 2007), the peculiar Tsingymantis antitra at Ankarana(Glaw et al. 2006), some Boophis and some cophyline microhylids (Köhler et al. 2007, Glaw et al. 2007) in the huge karstic massif of Tsingy de Bemaraha, and four large-bodied cave-dwelling species of Stumpffia from karstic regions in the North (Köhler et al. 2010).

During recent herpetofaunal inventories we discovered a further new species of a rather inconspicuous Gephyromantis frog inhabiting the deciduous forest of the karstic Bemaraha Plateau.

**Figure 1.**
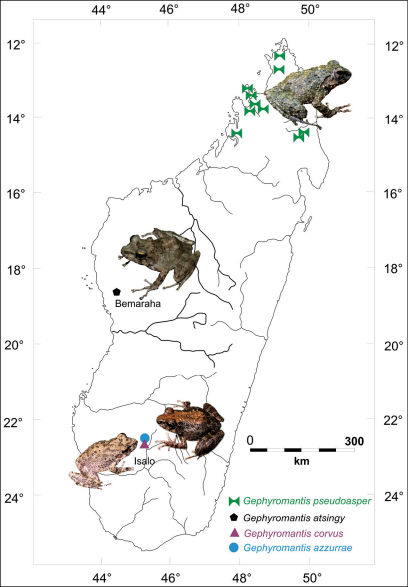
Schematic map of Madagascar with images and distribution of the four described species of the genus Gephyromantis, subgenus Phylacomantis.

Thirty-six described species are currently ascribed to Gephyromantis that is currently divided in five subgenera, including Phylacomantis. Four species are currently ascribed to this subgenus: Gephyromantis corvus Glaw & Vences, Gephyromantis pseudoasper Guibé, Gephyromantis azzurrae Mercurio & Andreone and the new species described in the present paper. With the exception of Gephyromantis pseudoasper, that mostly inhabits the rainforests of the North, the other species are found only in xeric habitats in the south-western (Gephyromantis corvus and Gephyromantis azzurrae) and western Madagascar (the new species described herein) (Fig. 1).

Due to morphological and external similarities, the new frog was formerly believed to be related to Gephyromantis corvus, a frog endemic of the Isalo Massif.

Unfortunately, the secretive life of this new species prevented us from obtaining much biological informations and we still lack information about its acoustic repertoire, breeding behaviour and larval morphology. Notwithstanding these challenges, the ongoing collaborative effort generated by the ACSAM (A Conservation Strategy for the Amphibians of Madagascar, Andreone and Randriamahazo 2008) allowed us to integrate the data and photographs obtained by three independent survey teams.

We present here the formal description of this new Gephyromantis species of the subgenus Phylacomantis, which differs from the other Phylacomantis species by a combination of morphological traits colouration and by a high divergence in mitochondrial DNA sequences.

## Methods

### Study site

The Tsingy de Bemaraha is a karstic plateau in the Melaky Region, five to 15 km wide and about 100 km long, located in western Madagascar. Numerous long, sharp pinnacles of rock, that may reach 45 meters in height, outcrop along the plateau and form the characteristic landscape (the so-called “tsingy” or “atsingy” in Malagasy language). Dry, deciduous forest is the most common vegetation type but humid areas occur within some of the larger canyons. An extensive area of forest and rock outcrop is included within two adjacent protected areas (Parc National Tsingy de Bemaraha and Réserve Naturelle Intégrale du Tsingy de Bemaraha). Savanna grasslands surround the plateau and there are numerous marshy depressions, caves and gorges associated with the main outcrop. This area has been object of some herpetological surveys that led to the discovery and description of several new species of amphibians (Vences et al. 2000, Glos et al.2005, Köhler et al. 2007, Glaw et al. 2007, Andreone and Randrianirina 2008) and reptiles (Schimmenti and Jesu 1996, Nussbaum and Raxworthy 2000, Glaw et al. 2009a, b).

### Sampling methods

We searched for frogs at night with the aid of hand torches and headlamps. Geographic coordinates were taken using a GPS device. Toponyms often follow the indications by local people, and must be therefore seen as largely unofficial names. Frogs were collected by hand and euthanised by immersion in chlorobutanol solution, fixed in 5% formalin or in 90% ethanol and finally stored in 75% ethanol solution. Voucher specimens (Tab. 1) are currently housed at the Museo Regionale di Scienze Naturali di Torino (MRSN), Zoologische Staatssammlung München (ZSM), and Université d’Antananarivo, Département de Biologie Animale (UADBA). Original field numbers are FN and FAZC (Franco Andreone Zoological Collection), FGZC (Frank Glaw Zoological Collection), BMR (Jasmin E. Randrianirina), and RBJ (Richard K. B. Jenkins). A few individuals do not bear any field number (no field number = NFN). The specimens of the type series were compared with the specimens of the other known species of the Phylacomantis subgenus (Tab. 1): six specimens of Gephyromantis azzurrae from Isalo, five specimens of Gephyromantis corvus from Isalo, and three specimens of Gephyromantis pseudoasper from Nosy Be (see Tab. 1). The comparative specimens of Gephyromantis pseudoasper were most probably temporary stored in denaturating solutions, and the sequencing was therefore not successful. For this reason, the three specimens here analyzed were only compared morphologically (Tab. 1), and the required sequences were retrieved from GenBank (DQ987513, DQ987515, DQ987517, DQ987518; DQ926890; AY848422-AY848424). Morphological information on Gephyromantis azzurrae specimens were taken from Mercurio and Andreone (2007) and sequences were retrieved from GenBank (EF222300- EF222305).

### Morphological measurements

Morphological measurements were made with a digital calliper to the nearest 0.1 mm. The following biometric measurements were taken (according to Mercurio and Andreone 2007): SVL (snout-vent length), HW (head width), HL (head length), ED (horizontal eye diameter), END (eye-nostril distance), NSD (nostril-snout tip distance), NND (nostril-nostril distance), TD (horizontal tympanum diameter), HAL (hand length), FORL (forelimb length), HIL (hind-limb length), FOL (foot length), FOTL (foot length including tarsus), IMTL (length of inner metatarsal tubercle), IMTH (height of inner metatarsal tubercle), FGL (length of the femoral macrogland cluster), FGW (width of femoral macrogland cluster). Webbing formulae follow Blommers-Schlösser and Blanc (1991), and femoral glands definition follows Glaw et al. (2000). For a few individuals we also counted the number (NG) and mean diameter (GD) of granules composing the right femoral gland. Granules were counted after having opened and flipped the gland.

### DNA analysis

A fingertip, or part of the muscle of the tongue, was cut from each collected individual and stored in 99% ethanol. Total genomic DNA was extracted from the tissue samples using proteinase K digestion (10 mg/ml concentration) following Bandi et al. (1994) protocol. To sequence a fragment of ca. 550bp of the mitochondrial 16S rRNA gene, which has proven to be suitable in anuran species identification (Vences et al.2005) we used the primers 16SA-L 5’-CGCCTGTTTATCAAAAACAT-3’ and 16SB-H 5’-CCGGTCTGAACTCAGATCACGT-3’, modified from Kocher et al. (1989) and Palumbi et al. (1991). PCR reactions were performed using standard cycling protocols (Vences et al. 2003) and the light strands were sequenced using an ABI3730XL by Macrogen Inc. Sequences were blasted in GenBank, checked by eye, edited, aligned using the BioEdit sequence alignment editor (version 7.0.5.3; Hall 1999). The alignment of all the processed samples required the inclusion of gaps to account for indels in only a few cases in one hypervariable region. All newly determined sequences have been deposited in GenBank (HQ640413-HQ640426). Mean genetic distances matrix (uncorrected *p*-distance transformed into percent) between and within individuals belonging to the type series of Gephyromantis atsingy (holotype and 7 paratypes) and of other species of the subgenus Phylacomantis (Gephyromantis corvus, Gephyromantis pseudoasper and Gephyromantis azzurrae) were computed.

## Results

### Gephyromantis (Phylacomantis) atsingy sp. n.

urn:lsid:zoobank.org:act:94995B02-B47C-4275-A6BA-DD4134B51203

Figures 2, A–L 

#### Etymology.

The specific noun “*atsingy*” (pronounced: “a-tseen-jě”) is a Malagasy word. The terms “atsingy” or “tsingy” are the common names used to refer to the pointed and sharp calcareous lime stone formations and pinnacles originated through rainfall erosion. Although present in several other localities in western Madagascar (e.g.: Ankarana), the outcrops of Bemaraha are typical of this area and the specific name is therefore associated with the locality of provenience of the types.

#### Remark.

This species has been referred to as Gephyromantis sp. aff. *corvus* “Bemaraha” by Glaw and Vences (2007), as Gephyromantis sp. 10 “Bemaraha” by Vieites et al. (2009), and Gephyromantis sp. aff. *corvus* by Bora et al. (2010).

#### Holotype.

MRSN A5487 (NFN), subadult male, collected at Tsingy de Bemahara National Park, western Madagascar, Andamozavaky (Bekopaka commune, Antsalova district, Melaky region, Mahajanga province), 19°01.86'S, 44°46.80'E; 122 m a.s.l., collected by J. E. Randrianirina on 23 May 2003.

#### Paratypes.

MRSN A5486 (BMR 001), subadult male without evident femoral glands, MRSN A5484 (NFN), adult female, MRSN A5482 (BMR 008), MRSN A5483 (BMR 031), MRSN A5485 (BMR 002), three juveniles (sex unknown) sampled from the same locality, collector and date of the holotype (tissue sample taken for genetical analysis for all individuals); ZSM 23/2006 (FGZC 0715), adult female, from Grotte Crystal, close to Andranopasazy, Tsingy de Bemaraha National Park (Antsalova commune, Antsalova district, Melaky region, Mahajanga province), 18°42'31"S, 44°43'08"E, 146 m a.s.l., collected by F. Glaw, J. Köhler, P. Bora and H. Enting on 19 March 2006, fixed in ethanol (tissue sample taken for genetical analysis), individual found at night on limestone cliffs, close to the entrance of the cave; ZSM 37/2006 (FGZC 0746), juvenile (unknown sex) from Grotte Anjohimbazimba, Tsingy de Bemaraha National Park (Antsalova commune, Antsalova district, Melaky region, Mahajanga province), 18°41'34"S, 44°42'36"E, 160 m a.s.l., collected by F. Glaw, J. Köhler, P. Bora and H. Enting on 20 March 2006 (tissue sample taken for genetical analysis), individual found in the cave; ZSM 107/2006 (FGZC 0886), juvenile (sex unknown) from Bendrao Forest (“Camp 3”), Tsingy de Bemaraha National Park (Antsalova commune, Antsalova district, Melaky region, Mahajanga province), 18°47'04"S, 44°51'37"E, 427 m a.s.l., collected by F. Glaw, J. Köhler, P. Bora and H. Enting on 26–27 March 2006; (tissue sample taken for genetical analysis). All these specimens were fixed in 90% ethanol and preserved in 70% ethanol. UADBA 28112 (RBJ 708), female from Ranotsara (Bekopaka commune Antsalova district, Melaky region, Mahajanga province), 19°02'08"S, 44°46'29"E, 65 m a.s.l., collected by R. Andriantsimanarilafy on 18 November 2006; UADBA 28116 (RBJ 792), female from Ankilogoa (Bekopaka commune, Antsalova district, Melaky region, Mahajanga province), 19°07'52"S, 44°48'32"E, 57 m a.s.l., collected by R. Randrianavelona on 13 December 2006; UADBA 28120 (RBJ 791), female from Ankilogoa (Bekopaka commune, Antsalova district, Melaky region, Mahajanga province), 19°07'52"S, 44°48'32"E, 57 m a.s.l., collected by R. Randrianavelona on 13 December 2006; UADBA 28127 (RBJ 718), female from Ranotsara (Bekopaka commune, Antsalova district, Melaky region, Mahajanga province), 19°02'08"S, 44°46'29"E, 65 m a.s.l., collected by R. Randrianavelona on 19 November 2006; UADBA 39057 (RBJ 660), female from Anjaha (Antsalova commune, Antsalova district, Melaky region, Mahajanga province), 18°39'43"S, 44°49'33"E, 403 m a.s.l., collected by J.C. Randrianantoandro, R. Randrianavelona, R.K.B. Jenkins, R.R. Andriantsimanarilafy and E.F. Hantalalaina and Madagascar National Parks personnel on 15–24 February 2006; UADBA 39081 (RBJ 609), female from Andranopasazy (Melaky region, Mahajanga province), 18°42'31"S, 44°43'02"E, 146 m a.s.l. collected by J.C. Randrianantoandro, R. Randrianavelona, R.K.B. Jenkins, R.R. Andriantsimanarilafy and E.F. Hantalalaina and Madagascar National Parks personnel on 13–30 January 2006; UADBA 39082 (RBJ 630), female from Andranopasazy (Antsalova commune, Antsalova district, Melaky region, Mahajanga province), 18°42'31"S, 44°43'02"E, 146 m a.s.l. collected by J.C. Randrianantoandro, R. Randrianavelona, R.K.B. Jenkins, R.R. Andriantsimanarilafy and E.F. Hantalalaina and Madagascar National Parks personnel on 13–30 January 2006; UADBA 39099 (RBJ 627), adult male (with developed glands) from Andranopasazy (Antsalova commune, Antsalova district, Melaky region, Mahajanga province), 18°42'31"S, 44°43'02"E, 146 m a.s.l. collected by J.C. Randrianantoandro, R. Randrianavelona, R.K.B. Jenkins, R.R. Andriantsimanarilafy and E.F. Hantalalaina and Madagascar National Parks personnel on 13–30 January 2006; UADBA 39100 (RBJ 658), female from Anjaha (Antsalova commune, Antsalova district, Melaky region, Mahajanga province), 18°39'43"S, 44°49'33"E, 403 m a.s.l., collected by J.C. Randrianantoandro, R. Randrianavelona, R.K.B. Jenkins, R.R. Andriantsimanarilafy and E.F. Hantalalaina and Madagascar National Parks personnel on 15–24 February 2006.

**Figure 2.**
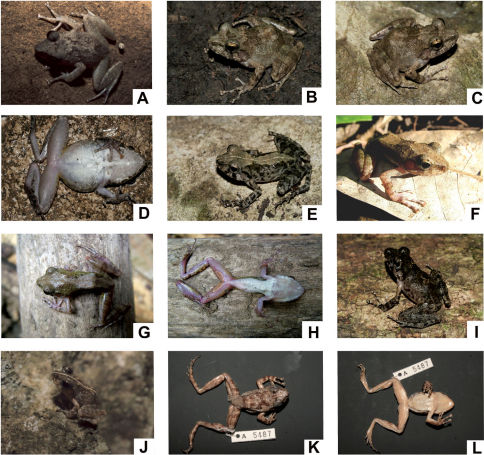
Images of Gephyromantis atsingy sp. n. **A** MRSN A5487 (NFN), subadult male (holotype) from Andamozavaky, dorsal view (photo by J. E. Randrianirina) **B–C** ZSM 23/2006 (FGZC 0715), adult female (paratype) from Grotte Crystal, close to Andranopasazy, dorsolateral views (photos by F. Glaw) **D** ZSM 23/2006 (FGZC 0715), adult female (paratype) from Grotte Crystal, close to Andranopasazy, ventral view (photo by F. Glaw) **E** ZSM 37/2006 (FGZC 0746), juvenile (paratype) from Grotte Anjohimbazimba (photo by F. Glaw) **F–G** UADBA 39099 (RBJ 627), adult male (paratype) from Andranopasazy, dorsolateral and dorsal views (photos by C. Randrianantoandro) **H** UADBA 39099 (RBJ 627), adult male (paratype) from Andranopasazy, ventral view, with evident and developed femoral glands of “Type 2” (photo by C. Randrianantoandro) **I** ZSM 107/2006 (FGZC 0886), juvenile (paratype) from Bendrao Forest (“Camp 3”), dorsolateral view (photo by F. Glaw) **J** MRSN A5483 (BMR 031), juvenile (paratype) from Andamozavaky, dorsolateral view (photo by J. E. Randrianirina) **K–L** MRSN A5487 (NFN), subadult male (holotype) from Andamozavaky, dorsal and ventral views of the preserved specimen.

#### Diagnosis.

A medium sized frog species (adult SVL 35–43 mm), assigned to the genus Gephyromantis (sensu Glaw and Vences 2006), subgenus Phylacomantis, according to genetic, phenetic and morphological similarities to the other known species (Gephyromantis azzurrae, Gephyromantis corvus, and Gephyromantis pseudoasper), and recognizable by the presence of the following characters:(a) femoral glands of “Type 2” (sensu Glaw et al. 2000), (b) webbing between toes present, (c) inner and outer metatarsal tubercles present, (d) tongue bifid, (e) lateral metatarsalia partly connected, (f) enlarged triangular finger tips, (g) not evident paired subgular vocal sacs, (h) crepuscular/nocturnal activity, (i) occurrence in limestone caves and deciduous forest habitat of dry western Madagascar.

**Table 1.** Morphometric measurements (in mm) of specimens of Gephyromantis atsingy, Gephyromantis corvus, Gephyromantis azzurrae and Gephyromantis pseudoasper. **HT** (holotype), **PT** (paratype). **M** (male). **F** (female). **J** (juvenile), **SMF** (Forschungsinstitut und Naturmuseum Senckenberg, Frankfurt, Germany). Other abbreviations are given in the text.

Catalogue number	Field number	Species	Locality	GenBank	Rank	SEX	SVL	HW	HL	ED	END	NSD	NND	TD	HAL	HIL	FORL	FOTL	FOL	IMTL	IMTH	FGL	FGW	NG	GD
MRSN A5487	NFN	G. atsingy	Andamozavaky	HQ640419	HT	M	34.8	14.1	14.5	5.7	3.8	2.6	3.6	3.3	11.6	37.5	18.6	24.8	17.7	1.1	1.1	-	-	5	0.7
MRSN A5486	BMR 001	G. atsingy	Andamozavaky	HQ640421	PT	M	31.3	12.4	13.5	5.3	3.9	1.9	3.1	2.8	10.6	33.1	16.2	23.6	15.5	1.7	1.2	-	-	-	-
MRSN A5484	NFN	G. atsingy	Andamozavaky	HQ640418	PT	F	43.4	15.9	17.3	6.4	4.5	3.1	3.9	3.6	13.3	43.2	20.1	30.9	20.4	2.2	1.0	-	-	-	-
ZSM 23/2006	FGZC 0715	G. atsingy	Grotte Crystal, close to Andranopasazy	HQ640414	PT	F	38.5	14.3	15.2	6.1	4.3	2.2	3.6	2.7	11.7	39.9	18.0	26.0	16.7	1.6	0.9	-	-	-	-
MRSN A5482	BMR 008	G. atsingy	Andamozavaky	HQ640420	PT	J	22.0	8.4	9.6	3.8	2.6	1.6	2.5	2.2	7.4	23.7	11.1	17.1	9.9	1.1	0.6	-	-	-	-
MRSN A5483	BMR 031	G. atsingy	Andamozavaky	HQ640417	PT	J	19.6	7.3	8.3	3.5	2.2	1.1	2.1	1.7	6.3	17.9	9.4	13.3	8.1	0.4	0.2	-	-	-	-
MRSN A5485	BMR 002	G. atsingy	Andamozavaky	HQ640415	PT	J	23.6	8.9	10.5	4.1	2.9	1.4	2.6	2.3	8.1	25.5	13.2	18.4	11.6	0.6	0.2	-	-	-	-
ZSM 107/2006	FGZC 0886	G. atsingy	Bendrao Forest	HQ640416	PT	J	22.5	7.8	9.5	3.1	2.4	0.9	2.1	1.8	6.5	22.4	10.9	16.5	10.0	1.0	0.3	-	-	-	-
ZSM 37/2006	FGZC 0746	G. atsingy	Grotte Anjohimbazimba	-	PT	J	24.6	8.2	10.1	3.2	2.7	1.5	2.3	1.9	6.9	24.2	11.1	17.1	10.8	1.1	0.5	-	-	-	-
UADBA 39099	RBJ 627	G. atsingy	Andranopasazy	HQ640413	PT	M	36.6	11.2	18.6	4.2	3.6	1.9	2.9	3.4	11.5	62.1	16.4	25.5	17.5	1.1	0.8	7.5	3.1	70	0.5
UADBA 39081	RBJ 609	G. atsingy	Andranopasazy	-	PT	F	38.4	14.8	16.8	4.3	3.6	1.9	3.4	3.3	11.9	63.5	17.4	27.3	19.3	1.4	0.8	-	-	-	-
UADBA 28120	RBJ 791	G. atsingy	Ankilogoa	-	PT	F	35.9	11.1	17.1	4.5	3.3	1.6	2.6	3.2	11.1	64.0	16.9	27.1	18.1	1.1	0.5	-	-	-	-
UADBA 39100	RBJ 658	G. atsingy	Anjaha	-	PT	F	35.1	12.1	17.8	4.4	3.9	1.7	2.7	3.3	11.0	62.2	17.3	26.8	17.3	1.3	0.7	-	-	-	-
UADBA 28127	RBJ 718	G. atsingy	Ranotsara	-	PT	F	39.1	12.7	17.9	4.6	3.7	1.6	3.2	3.3	12.1	64.0	18.4	28.4	18.7	1.4	0.6	-	-	-	-
UADBA 39082	RBJ 630	G. atsingy	Andranopasazy	-	PT	F	38.1	12.6	16.9	3.9	4.1	1.6	2.9	2.9	11.6	63.9	17.8	27.4	17.6	1.5	0.5	-	-	-	-
UADBA 28112	RBJ 708	G. atsingy	Ranotsara	-	PT	F	38.4	11.8	16.8	4.2	4.0	1.1	2.5	3.0	11.4	61.9	18	26.3	16.4	1.2	0.5	-	-	-	-
UADBA 39057	RBJ 660	G. atsingy	Anjaha	-	PT	F	35.3	11.4	17.4	4.3	3.6	1.4	2.9	2.8	11.6	63.9	16.6	27.5	18.4	0.9	0.5	-	-	-	-
UADBA 28116	RBJ 792	G. atsingy	Ankilogoa	-	PT	F	33.9	11.0	16.3	3.4	3.7	1.2	2.2	2.5	11.0	58.6	16.4	25.0	15.9	1.0	0.5	-	-	-	-
MRSN A5373	FAZC 12859	G. corvus	Isalo, Tsiombivositra	HQ640423	-	M	39.8	16.1	15.3	5.8	4.8	2.9	4.3	3.7	11.8	38.7	16.3	28.6	19.6	2.4	1.4	9.1	4.1	96	0.6
MRSN A5325	FAZC 13000	G. corvus	Isalo, Ambovo	-	-	F	40.0	15.2	16.2	6.4	4.3	2.6	4.1	3.2	10.7	40.3	18.2	30.1	20.0	1.6	0.6	-	-	-	-
MRSN A5323	FAZC 12661	G. corvus	Isalo, Malaso	HQ640422	-	F	39.0	15.0	15.7	6.1	4.4	2.8	4.2	3.6	11.9	39.3	17.6	29.7	20.3	1.6	0.8	-	-	-	-
MRSN A5324	FAZC 12758	G. corvus	Isalo, Zahavola	HQ640424	-	F	40.1	15.0	16.3	6.1	4.4	2.8	4.2	3.3	10.8	37.2	16.9	28.0	19.8	1.3	0.7	-	-	-	-
MRSN A2786	FAZC 11964	G. corvus	Isalo, Andranomena	HQ640425	-	F	40.8	15.0	15.8	6.3	4.7	2.7	4.3	3.6	11.1	40.5	17.7	30.2	20.4	1.9	0.7	-	-	-	-
MRSN A5310	FAZC 12568	G. azzurrae	Isalo, Andriamanero	EF222301	HT	M	41.1	16.9	13.4	6.1	3.9	2.5	4.0	4.0	12.1	41.1	20.0	30.0	18.8	2.0	1.1	6.3	2.0	45	0.5
MRSN A5309	FAZC 12567	G. azzurrae	Isalo, Andriamanero	EF222300	PT	M	38.5	15.3	12.8	5.2	4.3	2.2	3.7	3.7	11.1	41.1	19.9	26.7	18.9	1.9	1.3	6.5	2.7	38	0.5
MRSN A5311	FAZC 12569	G. azzurrae	Isalo, Andriamanero	EF222302	PT	M	40.2	15.8	14.1	6.0	4.0	2.7	4.0	4.1	11.2	41.0	19.9	27.7	19.9	2.1	1.1	6.7	2.7	40	0.6
MRSN A5312	FAZC 12910	G. azzurrae	Isalo, Iambahatsy	EF222304	PT	M	23.3	8.8	8.8	4.1	2.8	1.4	2.2	2.5	8.8	24.5	11.1	17.7	12.1	1.1	0.5	_	_	_	_
SMF 85859	NFN	G. azzurrae	Isalo, Sakamalio	EF222305	PT	M	42.7	16.4	14.3	5.4	3.7	2.6	3.9	3.5	13.4	41.1	21.0	29.9	20.0	1.9	1.1	7.0	3.0	42	0.6
SMF 85860	NFN	G. azzurrae	Isalo, Sakamalio	EF222303	PT	M	43.7	16.4	13.5	5.7	4.0	2.5	3.8	4.0	12.2	42.3	21.1	27.7	20.0	1.9	1.0	7.5	2.7	42	0.5
MRSN A3415	NFN	G. pseudoasper	Nosy Be	-	-	M	33.3	12.1	13	4.9	3.8	2.6	2.8	3.1	10.4	52.4	15.7	23	17.5	2.1	1.1	6.1	2.5	43	0.3
MRSN A3416	FN 6696	G. pseudoasper	Nosy Be	-	-	M	37.4	12.9	15	4.9	4.4	3	3.8	4.4	10.3	54.2	16.4	25	15.8	2.4	1.1	7.2	2.9	39	0.4
MRSN A3417	FN 6423	G. pseudoasper	Nosy Be	-	-	F	33.1	11.9	14	5.3	3.7	2.7	2.9	3	9.9	56.5	16.5	24	18.2	1.9	1	-	-	-	-

#### Description of the holotype.

Subadult male in mediocre state of preservation, with the belly opened for gonadal inspection and part of the ventral surface of thighs cut and opened to check the glands. SVL 34.8 mm; for other measurements see Tab. 1. Body slender; head longer than wide, in line with the body; snout slightly pointed in dorsal view, rather rounded in lateral view; nostrils directed laterally, much nearer to tip of snout than to eye; canthus rostralis well defined; tympanum distinct, rounded, its horizontal diameter about 50% of eye diameter; supratympanic fold well distinct, regularly curved; tongue distinctly bifid posteriorly. Arms slender; subarticular tubercles single; outer and inner metacarpal tubercles paired; fingers without webbing; finger disks triangular distinctly enlarged; nuptial pads absent. Hind limbs slender; tibiotarsal articulation reaching the nostril when hindlimbs are adpressed along body; lateral metatarsalia partly connected; inner metatarsal tubercle distinct, outer metatarsal tubercle small but recognizable; webbing of foot 1(1), 2i(1), 2e(1), 3i(2), 3e(1), 4i(2), 4e(2), 5(1). Skin slightly granular on dorsum and belly, ventral skin smooth on throat and chest. Femoral glands cluster ("Type 2", according to Glaw et al. 2000) hardly recognizable from external view, but with an overall granular structure and with 4–6 single whitish granular glands of ca. 1 mm diameter scattered on thighs. The vocal sacs in the male holotype are indistinct. The live colouration, based upon the photograph taken by J.E. Randrianirina is light brownish with darker dots and marbling (Fig. 2; A). The finger and toe tips are lighter than the remnant parts of fore- and hindlegs. After about seven years of preservation in ethanol the holotype still conserves the original marbled-brownish colour patterns, although it showed a slight loss of colour (Fig. 2; K–L). In particular, the belly became much whitish and inconspicuous. A rather characteristic and darker X-shaped marking is visible on the shoulder region, as well as a diffuse marbling darker pattern on the back and head. The tympanum is whitish. Limbs are brownish, with dark brown cross-bands: 3 on femur, 3 on tibia, 5–6 on tarsus and foot, 4 on lower arm and hand. On the flanks, the dorsal colour fades into the whitish ventral colour. The ventral side is uniformly cream-whitish on forelimbs and belly, while the throat is very lightly pigmented.

#### Variation.

We based the current description of variability upon some specimens (paratypes and complementary individuals), part of which (ZSM 23/2006, 37/2006, 107/2006, MRSN A5486 and MRSN A5483) were also photographed in nature, and thus provided more diagnostic characters. The female ZSM 23/2006 (Fig. 2; C–E), shows a back with sparse larger warts. Its colouration appears light brown with greyish shadings, darker dots and transversal bands on the back and legs. These are more evident in the preserved individual, where a pattern of darker spots is visible on the back, suggesting the presence of a darker X-shaped drawing. These spots are visible in two other individuals, MRSN A5484 (a female) and in the holotype MRSN A5487 (Fig. 2; A, K), although for the former specimen we do not have photographs taken in life. The tympanum is uniformly brownish, and the iris is yellowish with darker reticulations. The belly is comparatively smooth, with fewer warts on its lateral parts. The throat is quite smooth. The central part of the belly is lighter than the flanks and the ventral sides of thighs, whitish on breast and thorax, with sparse darker spots. The inguinal part appears yellowish. The throat is darker than the belly, with a median lighter (although not so contrasted) line. The lateral borders of the lower jaw bear darker spots. After preservation, the colouration appears substantially similar, although faded. The juvenile ZSM 37/2006 (Fig. 2; E) presents a rather smooth back and flanks with sparse and barely evident warts. The colouration is brownish shading to the grey on the flanks and lateral parts of the back, with darker spots, extending around the flanks. The central part of the back is crossed by a longitudinal light (almost beige) band which enlarges on the head to cover the upper eyelids. The posterior part of such a band narrows to shade almost totally at the level of the vent. A thin, almost continuous whitish longitudinal line runs from the tip of the snout until the groin. The juvenile ZSM 107/2006 (Fig. 2; I) also shows a rather smooth back. The colouration is much darker, and the markings and spots are less visible. The tympanum is lighter than the surrounding areas, and the upper ridge is entoured by black pigment. Both these juveniles after about four years of preservation present a similar pattern of colouration as in life. In ZSM 107/2006 the central part of the back appears quite lighter than the surrounding areas, with a sort of arrow pattern. An interesting comparison is with the only mature available male (SVL 36.6 mm) photographed in life, the individual labelled UADBA 39099 (Fig. 2; F–H). This male appears quite slim in the photographs (either in dorsal or ventral view), with rather uniform light brown shading to greenish in life, and a moderately glandular skin texture (Fig. 2; F–G). The belly appears rather smooth in life, with the whole venter and thorax whitish (Fig. 2; H). The throat is darker with a rather indistinct central whitish band and vocal sacs are not recognizable. Lower parts of arms and thighs are pinkish, while tibiae are more whitish pigmented. The plantar surfaces are also reddish-pink. In this male, the glands are well visible and yellowish, and appear similar to those observed in Gephyromantis azzurrae, Gephyromantis corvus and Gephyromantis pseudoasper. In particular, they clearly belong to the gland “Type 2”, sensu Glaw et al. (2000), with 70 granules counted from the inner side of the right gland itself (whose external measure is 7.5*3.1 mm). In MRSN A5484 (a female) we notice a dark bar between the eyes, and an X-shaped darker spot at mid-dorsum; quite large and isolated dark spots are visible in the posterior part of the back. The belly is uniformly whitish and smooth. The three juveniles MRSN A5483, MRSN A5482, and MRSN A5485, are similar in colouration (excepting for MRSN A5483 exhibiting a light mid-dorsal line), with dark back with sparse lighter spots and shading, and almost whitish bellies. Of MRSN A5483 we also dispose of a photo taken in life, where the longitudinal light line is evident (Fig. 2; J).

#### Natural history.

According to our observations, the species lives in habitats that retain some humidity, such as rock cavities and along the walls of the canyon-like formations. One important notation comes from the fact that several of the collectors, independently (JER, FG, JCR) found this species within the caves which are typical of the area. We suspect that the species uses caves because these sites presumably have a higher humidity than the surrounding areas. In such a sense it behaves similarly to Gephyromantis corvus at Isalo, which is known to frequent narrow canyons and cave-like canyons (Mercurio et al. 2008). Apparently, the new species (both adults and juveniles) is not confined to the proximity of water, and it has been observed jumping among the tsingy pinnacles also far from water bodies. All the individuals were active at night on tsingy rocks or during the day in caves. No data are available about mating behaviour, advertisement calls and tadpole morphology.

#### Distribution.

Only known from the localities of the type specimens within the Tsingy de Bemaraha National Park.

#### Comparison with other species.

Gephyromantis atsingy sp. n. differs from Gephyromantis pseudoasper, Gephyromantis azzurrae and Gephyromantis corvus by the lack of paired blackish skin folds (vocal sacs) along the lower jaws in adult males, and from Gephyromantis azzurrae also by details of colouration (see below). Following our measurements, adult males of Gephyromantis atsingy can also be differentiated among each other by the number of granules in the femoral glands: 70 granules in Gephyromantis atsingy; 96 granules in Gephyromantis corvus; 38–45 granules in Gephyromantis azzurrae and 39–43 granules in Gephyromantis pseudoasper. In addition, the new species differs from all three species by substantial genetic differentiation (see below).

All the described species of Gephyromantis, subgenus Phylacomantis, show similarities with Gephyromantis atsingy (Tab. 2). The dorsal pattern is similar in all species, showing an assemblage of darker spots and reticulations on the lighter background, and barred legs and arms. The dorsal colouration in Gephyromantis atsingy is usually light brown-beige, with a somehow greenish shading, while in Gephyromantis corvus it is uniformly grey or dark grey with sparse darker (uniformly-sized) warts and dots. Notwithstanding, the examined specimens of Gephyromantis atsingy have a much more contrasted X-shaped dark spot on the back. This is less evident in Gephyromantis corvus, where the dark-light pattern is more confuse and irregular. We observed a longitudinal repetition of lighter elements, a longitudinal light band or a middorsal light line only in Gephyromantis atsingy. The belly in both species is light, but in Gephyromantis atsingy we detected more frequently the darker drawing with a lighter central area on the throat and chest. According to the original description and subsequent papers (Mercurio and Andreone 2007, Mercurioet al. 2008), Gephyromantis azzurrae has a quite variable dorsal colouration. The holotype of the species, as depicted by Glaw and Vences (2007), has a wide lighter dorsal band upon a darker dorsal colouration, and the belly is reddish. Other examined specimens of Gephyromantis azzurrae present more uniform dorsal colouration. In both species the dorsal skin is featured by the presence of similar larger warts. In comparison to Gephyromantis atsingy, the Gephyromantis pseudoasper specimens are smaller and have a more warty back. The colouration in Gephyromantis pseudoasper is much darker and the belly is much more pigmented: the throat, the thorax and the anterior part of the belly are heavily spotted in dark, with a clear median light line on the throat. The posterior parts of the belly and parts of the ventral side of the legs in Gephyromantis pseudoasper are often orange. The external vocal sacs are evident and well developed, while these are not visible in Gephyromantis atsingy.

**Table 2.** Distribution, habitats and diagnostic characters of the nominal species in the genus Gephyromantis, subgenus Phylacomantis.

Species	Distribution	Habitat	SVL	Vocal Sacs	Dorsal colouration	Ventral colouration	Dorsal texture
Gephyromantis atsingy	Tsingy de Bemaraha	Karst pinnacles and caves	35–43 mm	Non-evident	Light brownish with greenish shadings	Whitish	Slightly warty
Gephyromantis azzurrae	Isalo Massif	Open canyons and permanent rivers	23–44 mm	Double and brownish	Brownish, sometimes with wide light band	Whitish, with reddish shadings	Warty with heterogeneous warts
Gephyromantis corvus	Isalo Massif	Close canyons and cave-like canyons	39–41 mm	Double and blackish	Greyish with darker spots	Whitish	Warty
Gephyromantis pseudoasper	Sambirano, N, NW and NE	Rainforests, karstic areas	33–37 mm	Double and blackish	Brownish	Whitish	Slightly warty

#### Mitochondrial variation and differentiation.

The molecular data confirm the attribution of Gephyromantis atsingy to the subgenus Phylacomantis (Glaw and Vences 2006, Vieites et al. 2009). The analyzed specimens of Gephyromantis atsingy, Gephyromantis azzurrae, Gephyromantis corvus and Gephyromantis pseudoasper appear genetically very uniform and show an intraspecific uncorrected divergence of 0.5%, 0.4%, 0.1% and 0.1% respectively, in the 16S rRNA gene sequences. The genetic distance between Gephyromantis atsingy and the three other Phylacomantis species ranges between 10.2% (comparison between Gephyromantis atsingy and both Gephyromantis corvus and Gephyromantis pseudoasper) and 11.2% (comparison between Gephyromantis atsingy and Gephyromantis azzurrae). Among the genus Gephyromantis the smallest genetic distance is observed between Gephyromantis corvus and Gephyromantis azzurrae (9.1%) and the highest uncorrected divergence between Gephyromantis azzurrae and Gephyromantis pseudoasper (13.1%). Gephyromantis corvus and Gephyromantis pseudoasper have a genetic distance of 12%. These divergences are comparatively high among mantelline species (see Vences et al. 2005, Vieites et al. 2009), and corroborate the species status of Gephyromantis atsingy. The phylogenetic relationships between the species of the Phylacomantis subgenus have been resolved recently (N. Kaffenberger et al., in preparation). These analyses confirm the monophyly of the subgenus, provide evidence for the basal position of Gephyromantis pseudoasper and uncover the sister relationship between Gephyromantis atsingy and the complex made of Gephyromantis corvus and Gephyromantis azzurrae.

#### Conservation.

This species appears to be restricted to the Bemaraha Plateau, where it has been found in seven localities within the Tsingy de Bemaraha National Park. It may also occur in the Réserve Naturelle Intégrale, which forms the northerly limit of the Bemaraha Plateau, but survey data are lacking from this site. Within the national park, some areas of forest are damaged by conversion to agriculture and charcoal production, but the humid canyons where G. atsingy occur are generally well protected. We therefore recommend assigning a category of Near Threatened because the species nearly qualifies for listing as Vulnerable under D2: the species is confined to a single site, the Bemaraha Massif (1,577 km2), with a plausible threat that could impact the species in the near future. If the threat became operational, the species would be eligible for listing as Endangered since its extent of occurrence is well within the 5,000km2 threshold under the B criterion and it would occur at a single location (where the threat is habitat loss from agricultural activities and charcoal production) and there would be a continuing decline in the quality and area of habitat, qualifying the species for the criteria B1ab(iii).

## Discussion

The Bemaraha plateau is one of the most peculiar areas of western Madagascar in terms of amphibian richness and endemicity (Raselimanana 2008, Bora et al. 2010). The new Gephyromantis species described here adds one more taxon to a list of endemics, which includes Heterixalus carbonei, Plethodontohyla fonetana, Rhombophryne sp., Stumpffia sp. aff. *helenae*, Boophis tampoka (Vences et al. 2000, Köhler et al. 2007, Glaw et al. 2007, Andreone and Randrianirina 2008, Bora et al. 2010), although some of these species might also be present at other sites of the West. The description of Gephyromantis atsingy, and the previously mentioned works underline how little we still know about the amphibian fauna of this part of Madagascar and stresses the importance of further systematic surveys in these isolated areas.

One powerful tool is the application of an integrated taxonomy approach, which includes aspects of direct field surveys, behavioural assessment, molecular screening and bioacustic analysis. This is what allowed Vieites et al. (2009) to identify a high number of candidate species boosting a descriptive process of a large number of poorly differentiated species in an astonishing short lapse of time (Mercurio and Andreone 2007, Cramer et al. 2008, Köhler et al.2008,^ 2010^, Wollenberg et al. 2008, Andreone et al. 2010, D’Cruze et al. 2010, Glaw et al. 2010, Vallan et al. 2010, Vences et al. 2010a, b).

As already stressed by Glaw and Vences (2007) and Bora et al. (2010), Isalo and Bemaraha were probably in contact until relatively recently and were covered by humid vegetation, which allowed the existence of typical rainforest species at Isalo (e.g., Boophis luteus, Mantidactylus femoralis and Mantidactylus lugubris), and rainforest-derived species at Bemaraha (e.g., Boophis tampoka, Plethodontohyla fonetana and Rhombophryne sp.). This hypothesis is also supported by the shared presence of typical rainforest elements, and by the presence of other species, like Mantella sp. aff. *expectata* and Blommersia sp. aff. *wittei*.

We expect that other forest blocks in western Madagascar may host further undescribed species of Gephyromantis and we highlight the need of conservation actions in Madagascar’s dry forests due to the increasing deforestation rate and changing climatic scenarios.

The accelerated species discovery in Malagasy amphibians points to the importance of taxonomic surveys (see www.sahonagasy.org), and we like to consider Gephyromantis atsingy as another “forceps delivered” species, according to the attractive definition given by Vallan et al. (2010) for Boophis calcaratus. In fact, we knew about the existence of Gephyromantis atsingy for several years, but did not have enough data to describe it, since Gephyromantis atsingy and Gephyromantis corvus appear to be rather similar to each other and share several life history traits. We here described this new species, recognizing that a species without a formal description and an attached name is simply an “invisible” species, hard to be protected and classified within the IUCN Red List (IUCN 2010).

## Supplementary Material

XML Treatment for Gephyromantis (Phylacomantis) atsingy
